# Hepatitis G Virus associated aplastic anemia: A recent case from Pakistan

**DOI:** 10.1186/1743-422X-8-30

**Published:** 2011-01-21

**Authors:** Shahida Amjad Riaz Shah, Muhammad Idrees, Abrar Hussain

**Affiliations:** 1 Hematology Department, Sheikh Zayed Medical Complex, Lahore Pakistan; 2 Division of Molecular Virology, National Centre of Excellence in Molecular Biology, 87-West Canal Bank Road Thokar Niaz Baig Lahore-53700, University of the Punjab Lahore, Pakistan

## Abstract

**Background:**

Aplastic anemia (AA) is a serious and rare disorder characterized by a hypocellular bone marrow. Hepatitis associated aplastic anemia (HAAA) is a variant of aplastic anemia in which aplastic anemia follows an acute attack of hepatitis. Several reports have noted an association between HGV and hepatitis-associated aplastic anemia besides other hepatitis causing viruses.

**Case presentation:**

A female girl of age 11 year with a history of loose motion for one month, vomiting for last 15 days and poor oral intake for last few days is reported here. The physical examination presents fever, pallor whereas bleeding, hepatomegaly, Splenomegaly and bruising were absent, abdominal ultrasonography confirmed the absence of hepatomegaly, Splenomegaly and lymphodenopathy. The laboratory investigation parameters were: haemoglobin 6.2 g/L, total leucocytes count 1.51, neutrophils 0.47%, absolute reticulocyte count 0.5%, Monocytes 0.16%, red cell count 3.2 mil/uL, Picked cell volume (PCV) 30.13%, Mean Corpuscular Volume (MCV) 78 fL, Mean Corpuscular Hemoglobin (MCH) 26.3 pg. The liver enzymes were alanine aminotransferease (ALT) 98 IU/L, aspartate aminotransferase (AST) 114 IU/L. Serologic and molecular tests for hepatitis A, B, C, D, E, TTV, B19 were negative, whereas HGV RNA PCR test was found positive for hepatitis G virus. The bone marrow aspirate and trephine biopsy examination revealed hypo- cellularity, erythropoiesis, myelopoiesis and megakaryopoiesis.

**Conclusion:**

HAAA is an uncommon but severe condition, which may occur following idiopathic cases of acute hepatitis. Our finding suggests the involvement of HGV in the development of aplastic anemia. In patients presenting with pancytopenia after an episode of acute hepatitis, the definitive diagnosis should be considered and confirmed by RT-PCR and if possible by bone marrow biopsy.

## Introduction

Hepatitis G virus was reported first time has a non-A-E hepatitis and placed as flavivirus [1].The induction of this new agent in the family of Hepatitis has attracted significant attention because of its etiology [2]. Hepatitis G virus has been marked as a cause of non-A through E acute viral hepatitis and sharp liver failure. Aplastic anemia complicating hepatitis is an uncommon but well recognized phenomenon. Hepatitis associated aplastic anemia is a severe disorder with a high mortality (85%) [3].Hepatitis associated aplastic anemia (HAAA) is a deviation of aplastic anemia in which aplastic anemia follows an acute attack of hepatitis. The marrow failure can be severe and is usually lethal if untreated. Lorenz and Quazier has documented first time HAAA in two case back in 1955 [4], by 1975 more than 193 cases had been reported [5]. Adil et al has reported, severe aplastic anaemia (SAA) 51.4%, very severe (VSAA) in 16.7% of 144 patients of aplastic anemia cases [6]. A number of reports have mentioned alliance between HGV and HAAA [7-10]. Number of HAAA cases with a history of multiple blood transfusions has been reported [11,12]. Crespo et al has documented a case of 24 year old man have community acquired HGV that later progress into severe aplastic anemia, point out HGV for both hepatitis and aplastic anaemia. However greater number of serum samples are needed to prove the association of hepatitis G virus and aplastic anaemia [13]. Moatter et al. has reported 5/43 patients of haemodialysis with raised liver enzyme, reduced platelet count and 21/100 patients of polytransfused b- thalassemia major children infected with HGV RNA form Pakistan [14,15].

## Case Presentation

In this study well characterized samples of 93 aplastic anaemia patients before blood tranfusion were included. These characteristics include history, physical examination, haematological investigation, bone marrow aspirate and trephine biopsy examination, liver function test (LFTs), renal parameters, viral profile and abdominal ultrasonography. The diagnosis of HA-aplastic anemia was made on the basis of hepatitis (elevated serum aminotransferase enzymes, jaundice, absolute neutrophils counts, platelet counts and reticulocytes. All the 93 samples were checked for serological marker of HAV, HBV,HCV, HDV, HEV, HGV,TTV and B19. One of 93 samples from patients with HA-aplastic anemia has hepatitis G associated aplastic anaemia with positive HGV RNA.

A female girl of age 11 year is reported here. The patient had a history of loose motion for one month, vomiting for last 15 days and poor oral intake for last few days. The physical examination presents fever, pallor whereas bleeding, hepatomegaly, Splenomegaly and bruising were absent, abdominal ultrasonography confirmed the absence of hepatomegaly, Splenomegaly and lymphodenopathy. The laboratory investigation parameters were: haemoglobin 6.2 g/L, total leucocytes count 1.51, neutrophils 0.47%, absolute reticulocyte count 0.5%, Monocytes 0.16%, red cell count 3.2 mil/uL, Picked cell volume (PCV) 30.13%, Mean Corpuscular Volume (MCV) 78 fL, Mean Corpuscular Hemoglobin (MCH) 26.3 pg. The liver enzymes were alanine aminotransferease (ALT) 98 IU/L, aspartate aminotransferase (AST) 114 IU/L. Serologic and molecular tests for hepatitis A, B, C, D, E, TTV, B19 were negative, whereas HGV RNA PCR test was found positive for hepatitis G virus. The bone marrow aspirate and trephine biopsy examination revealed hypo- cellularity, erythropoiesis, myelopoiesis and megakaryopoiesis.

## Discussion

Flaviviruses belong to enveloped viruses with a single positive sence RNA about 10 kb. Hepatitis G virus medium of transmission mostly through blood. The possible roel of hepatitis G virus infection in the pathogenesis of rare non-liver disease has been suggested but need to be recognized. By some unknow reseason aplastic anemia some time proceded by hepatitis. Few reports have analysed the role of HGV in the development of HAAA [8,9,11]. Crespo et al in print a case through negative serological markes for HAV, HBV, HCV, HEV, hypoplastic marrow low platelet and white cell counts but detected HGV-RNA before any blood transfusion [13]. Byrnes et al has described hepatit G Virus positive case of 26 year old man before the use of medication, blood tranfusion or intravenous drug abuse [9]. Zaidi et al, reported a 19 year male before blood transfusion with positive HGV by RT-PCR and suggested that in the absence of any other clincial manifestions the possible infectious agent may be HGV for hepatitis G virus associated aplast anaemia [8]. In the list of studies of Hepatitis G Virus associated aplastic anaemia before blood transfusion we report a case of 11 years female girls.

Whereas some reports confirms the presence of HGV viraemia in the patient aplastic anaemia after blood transufion[8,9,11,12,16-18]. But however concluded that HGV viraemia is frequent in patients with aplastic anaemia [19]. Kiem et al reported similar finding after logistic regression analysis, that HGV RNA in transfused patients was 5.9 times higher compare to untransfused patients (P = 0.001). This implicates transfusion as major source of HGV with aplastic anaemia [18].

The published literature point out that studies must be performed on many more aplastic anaemia patients prior to blood transfusion [20]. However, to find such patients in large number are not normally avaible to study, so far individual cases are reported. The ideal case regarding Hepatitis G associated aplastic anaemia are pre blood transfusion.

## Conclusion

In conclusion, HAAA is an uncommon but severe condition, which may occur following idiopathic cases of acute hepatitis. Our finding suggests the involvement of HGV in the development of aplastic anemia. In patients presenting with pancytopenia after an episode of acute hepatitis, the definitive diagnosis should be considered and confirmed by RT-PCR and if possible by bone marrow biopsy.

## Consent

Written informed consent was obtained from the patients a copy of which is available for review by Editor-in-Chief of this journal.

## Competing interests

The authors declare that they have no competing interests.

## Authors' contributions

SARS aided in acquistion of data that was included in this case report and drafted the manuscript. MI aided in acwuision and interpretation of the data analysed. AH helped in statistical analysis of the data and in editing of this manuscript. All authors have read and approved the final version of this manuscript.

## Authors Information

SARS: Consultant Hematologist, Sheikh Zayed Medical Complex. MI: Consultant Molecular Virologist, Ministry of Education. AH: Assistant Professor and PhD Research Scholar.
